# Clinical Reports

**Published:** 1859-11

**Authors:** 


CLINICAL REPORTS.—We propose hereafter to publish in every number of the Review a report of the more important and instructive cases in surgery that may fall under the observation of the senior editor at the clinic of the Jefferson College, at the Philadelphia Hospital, and in private practice. A gentleman well qualified for the task has been specially employed for the purpose.

